# Do biometric parameters improve the quality of optic nerve head measurements with spectral domain optical coherence tomography?

**DOI:** 10.1186/s12886-022-02281-6

**Published:** 2022-02-05

**Authors:** E. Elksne, J. V. Stingl, A. K. Schuster, F. M. Wagner, E. M. Hoffmann

**Affiliations:** 1 Department of Ophthalmology, Riga Stradins University, Dzirciema iela 16, Riga, 1007 Latvia; 2 Department of Ophthalmology, University Medical Center, Johannes Gutenberg University Mainz, Langenbeckstr. 1, 55131 Mainz, Germany

**Keywords:** SD-OCT, Optic nerve head, RNFL, MRW, BMO

## Abstract

**Background:**

Spectral domain optical coherence tomography (SD-OCT) is a widely applied non-invasive technique for evaluating optic nerve head parameters. The aim of this study was to evaluate the impact of biometric parameters such as the spherical equivalent (SE) and the anterior corneal curvature (ACC) on the peripapillary retinal nerve fiber layer (pRNFL), Bruch’s membrane opening (BMO), and the minimum rim width (MRW) measurements performed by spectral domain optical coherence tomography (SD-OCT) in glaucomatous and healthy eyes.

**Methods:**

In this cross-sectional, case–control prospective pilot study, the glaucoma group consisted of 50 patients with previously diagnosed and treated glaucoma and one healthy group of 50 subjects. Two consecutive examinations of pRNFL, BMO, and MRW with SD-OCT for every patient were performed without ACC and objective refraction (imaging 1) and with them (imaging 2).

**Results:**

The interclass correlation coefficient (ICC) reflected high agreement between imaging 1 and imaging 2 in both groups. The ICC in the glaucoma and healthy groups for pRNFL (0.99 vs. 0.98), BMO (0.95 vs. 0.97), and MRW (1.0 vs. 1.0) was comparable.

**Conclusions:**

Our preliminary data from a small number of eyes showed that the measurements of pRNFL, MRW, and BMO reflected high agreement between both imaging techniques with ACC and objective refraction and without these parameters in subjects with a refractive error up to ± 6.0 diopters. Further studies with participants with higher refractive error are necessary to evaluate the impact of biometric parameters such as SE and ACC on measurements with SD-OCT.

## Background

Spectral domain optical coherence tomography (SD-OCT) is a widely applied non-invasive technique for evaluating optic nerve head (ONH) parameters such as the peripapillary retinal nerve fiber layer (pRNFL), Bruch’s membrane opening (BMO), and the minimum rim width (MRW). The measurements provide a real-time in-vivo examination of ONH, and can also reflect the analysis of its progression, thus becoming an irreplaceable device in clinical practice [1–3].

Since the first articles about the application of optical coherence tomography at the end of the previous century [1, 4, 5], new technologies have significantly improved the reliability and quality of the device, resulting in higher scanning speed, increased axial resolution, and an eye-tracking system [6].

The Spectralis® SD-OCT (Heidelberg Engineering GmbH, Heidelberg, Germany) provides non-contact diagnostics with SD-OCT technology and is used for imaging the posterior segment of an eye. A super luminescence diode with an infrared beam central wavelength of 870 nm, which can acquire B-scans with a thickness of 11 µm. Furthermore, it provides a normative database of pRNFL thicknesses from healthy Caucasian subjects, leading to the possibility of performing quantitative analysis and comparing the results. Fovea-to-disc alignment technology can also improve the accuracy of ONH measurements [7].

Today, glaucoma is classified as the leading irreversible cause of blindness worldwide, with an increasing number of cases due to the aging population [8]. Pathogenesis of the disease relies on progressive loss of retinal ganglion cells (RGCs) and their axons [9]. Therefore, evaluation of ONH can provide clinically significant data about the status of the disease [10, 11]. Damage of ONH is the first sign of glaucomatous optic neuropathy and is only later followed by associated visual field defect, demonstrating the importance of a structural examination of ONH, especially during the early stages of the disease [12].

In a clinical setting, SD-OCT imaging is often performed without additional biometric parameters such as anterior corneal curvature (ACC) and refraction. Due to the increasing interest in the topic and the lack of publications in this field, the aim of this study was to evaluate the impact of biometric parameters such as spherical equivalent (SE) and ACC on the pRNFL, BMO, and MRW measurements performed by SD-OCT in glaucomatous and healthy eyes.

## Methods and materials

This cross-sectional case–control prospective pilot study was conducted at the University Medical Center Mainz. This study was established according to principles of the Declaration of Helsinki. This study was granted a waiver of ethical approval from the Medical Chamber of Rhineland-Palatinate, Germany. The data were analysed retrospectively.

Within the scope of the study, the patients were divided into two groups: The glaucoma group consisted of 50 patients with previously diagnosed and treated glaucoma and one healthy group of 50 subjects. Only one eye per patient was included. All patients underwent comprehensive ophthalmic examination to exclude any other pathology except refractive error and glaucoma.

Glaucomatous eyes were defined as glaucoma suspect optic discs (clinically and/or by optical coherence tomography with RNFL in the temporal superior or inferior segment out of normal limits), glaucoma suspect visual fields (VFs) with at least three consecutive examinations (one abnormal point below 0.5% on the pattern deviation plot or two adjacent points (cluster) beyond normal limits (*p* < 5%), and at least one point of them worse than 1% pattern deviation plot or three or more clustered points worse than 5% on the pattern deviation plot), and/or IOP (intraocular pressure) > 21 mmHg.

Healthy eyes did not have a history of increased IOP. The eye examination did not reveal any ophthalmic pathology, IOP was 21 mmHg or below, while VF was within normal limits.

The criteria for inclusion in this study were the following: Patients aged above 18 years, visual acuity (VA) LogMAR 0.5 or better, spherical equivalent in the range of –6.0 to + 6.0 D (diopters), and no history of previous corneal surgery. The exclusion criteria included a mean deviation (MD; dB) in the visual field for glaucoma patients of more than –10.0 dB, pseudophakia, diabetes, retinal photocoagulation, and any retinal or neurological diseases. Only one eye per patient was selected to be included, as pRNFL, BMO, and MRW show high correlation between both eyes [13].

Refractive error was derived from an objective refraction (Nidek AR1s, Nidek) based on an average of five readings. Ocular biometry was performed with Zeiss IOL Master 700 (Carl Zeiss Meditec AG, Jena, Germany) that provided axial length (AL) and anterior corneal curvature (ACC) values.

### Optical coherence tomography

BMO, MRW, and pRNFL were evaluated by SD-OCT (Spectralis® SD-OCT, Heidelberg Engineering GmbH, Heidelberg, Germany) and the associated Heidelberg Eye Explorer (version 1.9.14.0; HEYEX, Heidelberg, Germany) software. The eye tracking system was applied to automatically recognize the macula and optic discs; no manual corrections were carried out. The pRNFL values were derived from circumpapillary SD-OCT scans with a diameter of 4.1 mm.

We evaluated two consecutive examinations of pRNFL, BMO, and MRW with SD-OCT for every eye, carried out for quality assurance (Table 1). During the first imaging, no correction of ocular magnification was applied, except manual adjustment of the infrared image by focus correction. The default anterior corneal curvature value (7.7 mm) was applied. The second examination was characterized by manual adjustment of the infrared image. Additionally, the value of ACC and objective refraction were inserted into the HEYEX software for the correction of ocular magnification. Segmentation of pRNFL, BMO, and MRW was conducted by the application of HEYEX software.

**Table 1:** Examinations and applied correction of ocular magnification

Number of examinations	Manual adjustment of infrared image by focus correction	Anterior corneal curvature (ACC)	Objective refraction
Imaging 1	X		
Imaging 2	X	X	X

No dilating eye drops were applied prior to the examination. All scans were performed by two operators with experience in SD-OCT imaging. For quality control, all scans were manually reviewed and only high-quality, centered images with a signal strength above 25 dB were included, as recommended in the manufacturer’s guidelines.

### Statistical methods

For quantitative variables of normal distribution, the mean and standard deviation were reflected. For categorical variables absolute and relative frequencies were displayed. Chi-square tests and *t*-tests were computed. The intraclass correlation coefficient (ICC) was calculated to compare the readings of both methods of imaging. This was an explorative study; therefore, *p*-values are reported exactly. The data were analyzed using statistical software (IBM Corp. Released 2016. IBM SPSS Statistics for Macintosh, Version 24.0; Armonk, NY, USA).

## Results

Within the scope of this pilot study study, 50 eyes of 50 patients were examined in each group. Table 2 demonstrates the patients’ characteristics in both groups.

**Table 2:** Patients’ profiles in both study groups. BCVA, best-corrected visual acuity; IQR, interquartile range; D, diopter; SE, spherical equivalent; ACC, anterior corneal curvature; AL, axial length; SD-OCT, spectral domain optical coherence tomography; pRNFL, peripapillary retinal nerve fiber layer; BMO, Bruch’s membrane opening; MRW, minimum rim width). ^a^ Median was calculated

	**Glaucoma group** **(*N* = 50)**	**Healthy group** **(*N* = 50)**	***P***
**Sex**			0.69
Female	25 (50%)	27 (54%)	
Male	25 (50%)	23 (46%)	
**Age (years)**	49.2 ± 15.4	42.2 ± 17.8	0.02
**Eye**			0.83
Right	35 (70%)	34 (68%)	
Left	15 (30%)	16 (32%)	
**BCVA (LogMAR) **^**a**^	0.08 (IQR 0.10)	0 (IQR 0.06)	0.05
**SE (D)**	-0.69 ± 2.73 (range, -4.75 to + 3.36)	-0.46 ± 2.50 (range, -6.0 to + 6.0)	0.32
Sphere (D)	-0.31 ± 2.73	-0.14 ± 2.44	0.17
Cylinder (D)	-0.76 ± 0.76	-0.66 ± 0.53	0.21
**ACC (mm)**	7.76 ± 0.24 (range, 7.29–8.35)	7.77 ± 0.29 (range, 7.22–8.40)	0.91
**AL (mm)**	23.92 ± 1.29 (range, 19.62–26.61)	23.72 ± 1.14 (range, 21.67–27.08)	0.42
**SD-OCT**			
pRNFL (µm)	65.34 ± 17.17	82.70 ± 9.01	< 0.01
BMO (mm^2^)	1.93 ± 0.37	1.86 ± 0.32	0.33
MRW (µm)	234.74 ± 81.31	317.38 ± 56.88	< 0.01

The glaucoma group (the first group) consisted of 25 females and 25 males with a mean age of 49.2 ± 15.4 years, while the healthy group (the second group) included 27 females and 23 males with a mean age of 42.2 ± 17.8 years (*p* = 0.02). Visual acuity was slightly better in the second group (healthy group) (median LogMAR 0.08 (IQR 0.10) vs. 0 (IQR 0.06).

Biometric parameters such as SE (-0.69 ± 2.73 D vs. -0.46 ± 2.50 D; *p* = 0.32), ACC (7.76 ± 0.24 mm vs. 7.77 ± 0.29 mm, *p* = 0.91), AL (23.92 ± 1.29 mm vs. 23.72 ± 1.14 mm; *p* = 0.42), and BMO (1.93 ± 0.37 mm^2^ vs. 1.86 ± 0.32 mm^2^; *p* = 0.33) were comparable between both groups.

**Fig. 1:**
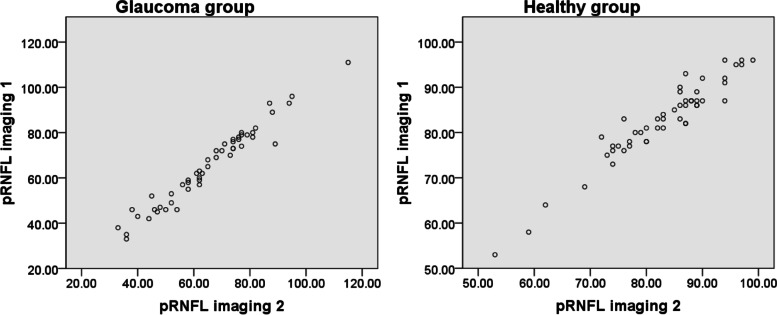
The correlation of the global pRNFL in each group between both imaging techniques. pRNFL, peripapillary retinal nerve fiber layer

The interclass correlation coefficient (ICC) reflected high agreement between imaging 1 and imaging 2 in both groups, with the highest value for MRW. Table 3 demonstrates all of the details of the examinations. No statistically significant difference was found between imaging 1 and imaging 2 for the healthy and glaucomatous eyes. Fig. 1 displays a scatter plot for global pRNFL in both groups.

**Table 3:** ICC for all examinations in the glaucomatous and healthy groups; *p*-value describes the difference between imaging 1 and imaging 2 in each group. ICC, interclass correlation coefficient; CI, confidence interval; BMO, Bruch’s membrane opening; MRW, minimum rim width**,** pRNFL, peripapillary retinal nerve fiber layer; G, global; TS, temporal superior sector; T, temporal sector; TI, temporal inferior sector; NI, nasal inferior sector; N, nasal sector; NS, nasal superior sector

	**Glaucoma group** **(*N* = 50)**		**Healthy group** **(*N* = 50)**	
	**ICC** **(95%CI)**	***P***	**ICC** **(95%CI)**	***P***
**BMO**	0.95 (0.92; 0.97)	**0.55**	0.97 (0.94; 0.98)	**0.44**
**MRW**	1.0 (0.99; 1.0)	**0.28**	1.0 (0.99; 1.0)	**0.48**
**pRNFL (G)**	0.99 (0.98; 0.99)	**0.95**	0.98 (0.96; 0.99)	**0.55**
TS	0.98 (0.97; 0.99)	**0.30**	0.96 (0.93; 0.98)	**0.60**
T	0.92 (0.86; 0.96)	**0.14**	0.98 (0.97; 0.99)	**0.75**
TI	0.99 (0.99; 1.0)	**0.60**	0.98 (0.96; 0.99)	**0.60**
NI	0.97 (0.94; 0.98)	**0.89**	0.98 (0.97; 0.99)	**0.08**
N	0.99 (0.97; 0.99)	**0.69**	0.97 (0.94; 0.98)	**0.88**
NS	0.97 (0.94; 0.98)	**0.17**	0.98 (0.97;0.99)	**0.92**

**Fig. 2:**
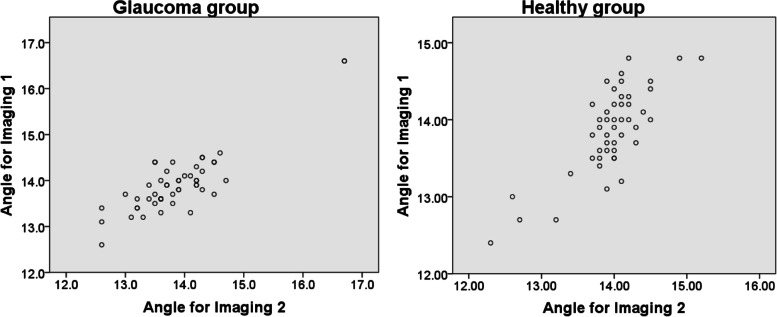
The correlation of the angle in each group between imaging techniques

Comparing the field of view (angle) for acquisition of the image between techniques, a high interclass correlation was seen in the glaucoma (ICC, 0.91; 95%CI, 0.83, 0.945) and healthy (ICC, 0.87; 95%CI, 0.78, 0.93) groups, as reflected in the Fig. 2. The median angle for imaging 1 (13.9 °, IQR 0.5 °) and imaging 2 (13.8 °, IQR 0.7 °) did not reveal a statistically significant difference (*p* = 0.46) in the glaucoma group. The same pattern was also observed in the healthy group (median 14.0 ° and IQR 0.4 ° vs. 13.9 ° and IQR 0.7 °; *p* = 0.12).

To increase the number of patients with medium-to-high refractive error, only patients with an SE + 2.0 or above and –2.0 or less were selected from both groups. In total, 34 eyes were included (21 with glaucoma and 13 healthy) with a mean SE of –1.86 ± 4.05 D and an ACC of 7.79 ± 0.27 mm. Statistical analysis did not reveal a significant difference between either imaging technique in terms of the results of BMO (ICC, 0.97; *p* = 0.51), MRW (ICC, 1.0; *p* = 0.91), and pRNFL (ICC, 1.0; *p* = 0.41).

## Discussion

The present study described the impact of biometric parameters such as SE and ACC on the measurements of BMO, MRW, and pRNFL using SD-OCT imaging.

SD-OCT is a common examination technique in daily practice and is frequently performed without biometric parameters, which could affect ocular magnification and the outcomes of measurements. Healthy participants and glaucoma patients were included in the present study to observe differences regarding ONH status. The glaucoma group included patients with early or moderate disease with good visual acuity, as significant damage of ONH could impact the reliability of the measurements due to the “floor effect” [14]. There was no significant difference between the participants of either group regarding sex, SE, ACC, AL, and BMO. The patients representing the glaucoma group were slightly older than those from the healthy group.

No statistically significant difference between either imaging technique was observed. BMO, MRW, and pRNFL were comparable between both methods in the glaucomatous and healthy eyes. Within the scope of this study, the correction of ocular magnification with ACC and SE did not improve imaging quality. Objective refraction showed a high correlation with the focus for infrared image in both groups, the glaucoma (ICC, 0.81; 95%CI, 0.68–0.90) and healthy (ICC, 0.94; 95%CI, 0.87–0.97) subjects. The focus for infrared image was applied in the used version of the Heidelberg Eye Explorer software to adjust for ocular magnification, if no further readings were inserted.

The results of this study could be explained by the fact that homogenous Caucasian groups with low refractive error in glaucoma and healthy eyes (SE, –0.69 ± 2.73 D vs. –0.46 ± 2.50 D) were selected and that the corneal curvature did not show a large variation in this population. Furthermore, a subgroup consisting of 34 patients with a higher level of SE (–1.86 ± 4.05 D) did not reveal a significant difference between either imaging technique regarding the results of BMO (ICC, 0.97; *p* = 0.51), MRW (ICC, 1.0; *p* = 0.91), and pRNFL (ICC, 1.0; *p* = 0.41). As the patients were included on a random basis according to their visits at the ophthalmology clinic, the study did not involve a significant number of participants presenting very high refractive errors. The outcomes related to SE could be explained by the fact that SD-OCT provides a correction of the subject’s refractive error for every scan by manual adjustment of the infrared image. In addition, the mean ACC value in both groups was similar to the default data in the HEYEX software. Therefore, the results do not fully describe the impact of ACC variations. A higher number of patients with significant differences in the standard ACC and SE values would be necessary.

For the performance of data analysis, the pRNFL values were derived only from circumpapillary SD-OCT scans with a diameter of 4.1 mm. The concentric circumpapillary scans with a diameter of 3.5 and 4.7 mm were not included in this study, as they provide complimentary information and show comparable diagnostic performance [15]. Furthermore, no patients with large areas of peripapillary atrophy were included.

SD-OCT could help in observing structural changes in ONH at an earlier stage. A previous study by Wesser et al. concluded that the progression rate in glaucomatous eyes is faster than in healthy eyes when measured by pRNFL loss [16]. Retinal ganglion cell axons can be evaluated by pRNFL and Bruch’s membrane opening-based MRW. The measurements of MRW demonstrate the minimum distance from BMO to internal limiting membrane, and together with pRNFL increase the diagnostic value for glaucoma [17]. Besides pRNFL and MRW, in the present study, BMO was evaluated using both techniques in order to compare the repeatability of measurements. Various publications have reflected that AL and SE are important factors to be accessed, while the pRNFL thickness profile could be shifted toward nasal or temporal sectors and could affect positive or negative glaucoma diagnosis [18–22].

Ocular magnification is affected by several parameters: AL, lens power and position, and corneal power. Earlier studies have demonstrated that ocular magnification is important for measurements made by OCT [23–26]. This is supported by the fact that reduced AL provides magnified ONH, while increased AL minified ONH [27]. The same applies to ACC that affects corneal power. Therefore, measurements of pRNFL are performed at different distances from the margin of ONH [28]. The size of ONH impacts the density of pRNFL—at the margin of ONH, it is thicker than for small ONH when compared with the fixed scan size, while for all optic nerve discs, the pRNFL thickness is lower when increasing the distance from margin of ONH [29, 30]. Consequently, corrected ocular magnification could reflect more reliable data and is very important when evaluating patients with glaucoma.

Spectralis® SD-OCT provides modified ocular magnification for all examinations to neutralize induced magnification automatically and generates individual length of scan according to refractive error, ACC, and non-changeable AL. First, it includes a pre-set AL of 24.385 mm and an ACC of 7.7 mm according to Gullstrand schematic eye. Furthermore, the device provides an option to change the ACC parameter as well. Second, it provides a possibility to focus the retinal image and, thus, to correct the patient’s refractive error [31, 32]. Company recommends using individual value of ACC for every examination since extreme values could influence the results (personal communication with Heidelberg engineering 2022). In addition, 0.1 mm error of ACC will induce about 0.8% error in lateral measurement. Ctori et al. concluded that performing area and lateral measurements parallel to the retinal surface, the fundus image focus according to refractive error and individual values of ACC should be included in order to correct the ocular magnification more precisely [32]. Scaled measures of RNFL is not related to axial length when ocular biometry is implemented [33].

In the present study, ocular magnification was corrected by individual values of ACC, SE, and manually adjusted infrared images by focus correction (bringing confocal scanning laser ophthalmoscopy images into sharp focus). The statistical analysis did not reveal any significant differences between imaging with and without biometric parameters such as ACC and SE. The default value of ACC in HEYEX software is 7.7 mm, which happened to be very similar to all of our patients’ mean values across study groups (7.76 ± 0.24 vs 7.77 ± 0.29 mm). SD-OCT provides an option to adjust the infrared image manually for every scan correcting the refractive error, however, ACC is not changed during manual adjustment. Furthermore, precise value of ACC could give correct magnification of the image and location of the scan. Lack of publications related to impact of objective parameters like SE and ACC on the outcomes of SD-OCT was a reason to perform the study.

In this cross-sectional, quality control audit, the authors did not succeed in establishing any significant differences between measurements of pRNFL, MRW, and BMO when evaluating the impact of ACC and SE among healthy patients and those suffering from glaucoma.

## Conclusions

In conclusion, our preliminary pilot study data from a small number of eyes showed that measurements of pRNFL, MRW, and BMO reflected high agreement between both imaging techniques with ACC and objective refraction and without these parameters in subjects with a refractive error up to ± 6.0 diopters. Further studies with participants with higher refractive error are necessary to evaluate the impact of biometric parameters such as SE and ACC on measurements with SD-OCT.

## Data Availability

The datasets used and/or analysed during the current study are available from the corresponding author on reasonable request.

## Abbreviations

SD-OCT: Spectral domain optical coherence tomography
ONH: Optic nerve head
pRNFL: Peripapillary retinal nerve fiber layer
BMO: Bruch’s membrane opening
MRW: Minimum rim width
RGC: Retinal ganglion cells
ACC: Anterior corneal curvature
SE: Spherical equivalent
RNFL: Retinal nerve fiber layer
VF: Visual field
IOP: Intraocular pressure
VA: Visual acuity
MD: Mean deviation
dB: Decibel
AL: Axial length
ICC: Interclass correlation coefficient
D: Diopter
BCVA: Best corrected visual acuity
IQR: Interquartile range
CI: Confidence interval
